# Case of Abstinence for Three Months, No Food, during That Period, Being Taken into the Stomach

**Published:** 1853-03

**Authors:** 


### ECLECTIC DEPARTMENT,

### AND SPIRIT OF THE MEDICAL PERIODICAL PRESS.

The following case is one of great interest and value, provided the reader can feel assured of the credibility of the narrative. We do not, of course, doubt the honesty of the reporter, but the account is so extraordinary, that a due regard to caution cannot but suggest the possibility, if not probability that the medical attendant was deceived by the patient as respects complete abstinence for the space of three months. Instances of a perverseness of human nature leading to similar acts of deception, are unfortunately but too numerous. We copy the account, and our readers will judge for themselves whether to regard it as containing facts having most important bearings on physiology and medical practice, or an illustration of human weakness and depravity.—EDITOR BUFFALO MED. JOUR.

*Case of Abstinence for three months, no Food, during that period, being taken into the stomach.* By J. L. PIERCE, M. D., of Fallsington, Bucks County, Pennsylvania.

On the 13th of January, 1836, my friend, Dr. May, was about leaving Philadelphia for the East, and requested me to take charge of a number of cases of considerable interest, which he then had under his care. Among these was a lady, who had for many months been severely affected with disease of the stomach. As the exact character of the disease may admit of dispute, and as it is not this to which I wish to draw attention, but a peculiarity in the mode of treating it, I have headed the article in the manner it stands.

I called, with Dr. May, to see this patient on the 13th of January, 1836. She was about 26 years of age, sallow complexion, emaciated, pulse 120, feeble, but regular. A spot over the left epigastric region, of an inch and a half in diameter, was very tender on pressure, and considerable tenderness existed for some distance around it. Her appetite, though not craving, was sufficiently good to enable her to eat almost any food, though she had been restricted to a very simple diet. Her bowels were open every two or three days, passages natural. She was in the habit of vomiting two or three times a day, and her food was generally rejected within a few minutes after it was taken. She had been sick about three months, and had been confined to the bed a large portion of this time.

I was informed that her case had been considered as carcinoma of the stomach. Her treatment had consisted of such articles as were calculated to allay the irritability of the stomach and the vomiting, and to quiet the severe pains of the epigastric region. A constant sore had been kept up over the affected spot by means of blisters, which were dressed with anodyne ointments, and occasionally leeches were applied to the spot.

On taking charge of the case, I watched it for some days very closely. I found that neither food nor medicine appeared to remain upon the stomach, but that as often as either was administered, vomiting ensued; and with the food ejected, there was from a tea-spoonful to a table-spoonful of matter, such as is usually discharged from an abscess. Should nothing be taken into the stomach for some hours, emesis would take place of this kind of substance by itself. During the first week, I pursued the same mode of treatment as had been made use of formerly, but I soon became satisfied that it was altogether useless, and that the patient must sink unless some more effectual plan was adopted. The simplest medicine taken into the stomach was rejected as soon as the severer kind, and even gum-arabic water or barley water was as irritating as oysters or roast beef could have been.

Toward the close of January, I asked her if she was willing to submit for one month to an entire abstinence of all kinds of food by the mouth, and be nourished solely by enemata. The idea was a novel one to myself, but my view was that the stomach must rest, and let the character of the disease be what it might, it would thereby stand a much better chance of healing. That there was a suppurating sore in the stomach appeared evident, and rest was absolutely essential. She was now exceedingly emaciated, and so feeble that she was entirely confined to the recumbent posture.

On the 1st day of February we entered upon our new mode of treatment. I directed lamb or mutton broth, of good quality, to be kept constantly on hand, and a half a pint to be used as an enema every three hours. During the first week, I allowed her to take not exceeding a tea-spoonful at a time of gum-arabic water or pure water, several times a day, with the request that she would lessen the frequency of it toward the latter part of the week, so as to be able to refrain from it entirely on the commencement of the second week. She very readily acceded to my wishes in every respect, and I have not the least doubt of her acting with perfect honesty toward me. I kept up a running sore over the epigastric region, which I dressed with simple cerate or basilicon ointment, sprinkled with morphia.

During the first week my patient vomited from three to six times daily, discharging pus, and a considerable quantity of matter resembling tubercles. Her sensation of hunger was at times great, but she bore it with remarkable fortitude. At the expiration of the week she was evidently more comfortable, and became increasingly so during each succeeding week of the month, at the close of which her condition was as follows: Vomiting occurs on an average about twice a day; substance discharged the same as before, tinged with a little blood; tongue clean; pulse fuller than formerly, and less frequent; countenance more healthy. I now asked her if she were willing to continue the same treatment for another month. She did not hesitate in giving a prompt response; and during this month, not a drop or morsel of any thing passed her lips.

*April* 1. Condition in every way improved. The vomiting occurs once in two or three days; quantity less; appearance of discharge white, with but very few of those particles resembling tubercles; bowels regular; pulse 90; very little pain; strength improving.

I now requested her to continue the treatment one month longer. She consented to do so, and it was astonishing how rapidly the improvement progressed. By the middle of the month I considered the cure to have been nearly completed, but thought it best not to depart from the course we had been pursuing, lest irritation of the stomach should renew the vomiting, and other unpleasant symptoms. About the 24th of the month, I allowed her a tea-spoonful of gum-arabic water three or four times a day, with directions to increase it in frequency each successive day, so that the stomach might be gradually prepared for its usual nourishment. By the close of the month I felt satisfied that the disease of the stomach had ceased, that the sore had healed, and that my patient, by a forbearance and perseverance seldom equaled, had been rescued from an untimely grave. She was now able to sit up a considerable portion of the day, a part of the time attended to some needle work or knitting; could walk some distance without assistance; complexion good; pulse 80; bowels regular, and much fleshier than when I commenced my attendance upon her.

I continued calling on the patient occasionally, until the 18th of May, when I took my leave of her, with many blessings showered upon my head.

In consequence of her removal to Southwark, I lost sight of this patient for nearly two years, when I accidentally met her in the street. Her appearance had so much improved that I scarcely recognized her, and she assured me that she was in the enjoyment of uninterrupted health.—*Amer. Jour. Med. Science.*

